# Gestational Heat Stress Alters Postnatal Offspring Body Composition Indices and Metabolic Parameters in Pigs

**DOI:** 10.1371/journal.pone.0110859

**Published:** 2014-11-10

**Authors:** Rebecca L. Boddicker, Jacob T. Seibert, Jay S. Johnson, Sarah C. Pearce, Joshua T. Selsby, Nicholas K. Gabler, Matthew C. Lucy, Timothy J. Safranski, Robert P. Rhoads, Lance H. Baumgard, Jason W. Ross

**Affiliations:** 1 Department of Animal Science, Iowa State University, Ames, Iowa, United States of America; 2 Division of Animal Sciences, University of Missouri, Columbia, Missouri, United States of America; 3 Department of Animal and Poultry Sciences, Virginia Tech University, Blacksburg, Virginia, United States of America; The University of Manchester, United Kingdom

## Abstract

The study objectives were to test the hypothesis that heat stress (HS) during gestational development alters postnatal growth, body composition, and biological response to HS conditions in pigs. To investigate this, 14 first parity crossbred gilts were exposed to one of four environmental treatments (TNTN, TNHS, HSTN, or HSHS) during gestation. TNTN and HSHS dams were exposed to thermal neutral (TN, cyclical 18–22°C) or HS conditions (cyclical 28–34°C) during the entire gestation, respectively. Dams assigned to HSTN and TNHS treatments were heat-stressed for the first or second half of gestation, respectively. Postnatal offspring were exposed to one of two thermal environments for an acute (24 h) or chronic (five weeks) duration in either constant TN (21°C) or HS (35°C) environment. Exposure to chronic HS during their growth phase resulted in decreased longissimus dorsi cross-sectional area (LDA) in offspring from HSHS and HSTN treated dams whereas LDA was larger in offspring from dams in TNTN and TNHS conditions. Irrespective of HS during prepubertal postnatal growth, pigs from dams that experienced HS during the first half of gestation (HSHS and HSTN) had increased (13.9%) subcutaneous fat thickness compared to pigs from dams exposed to TN conditions during the first half of gestation. This metabolic repartitioning towards increased fat deposition in pigs from dams heat-stressed during the first half of gestation was accompanied by elevated blood insulin concentrations (33%; P = 0.01). Together, these results demonstrate HS during the first half of gestation altered metabolic and body composition parameters during future development and in biological responses to a subsequent HS challenge.

## Introduction

Heat stress (HS) experienced *in utero* and during early development alters growth, behavior, learning capacity, body temperature and metabolic function that extend into post-gestational life [1]–[4]. Importantly, the timing and duration of gestational hyperthermia influences the severity of fetal and postnatal abnormalities [5], [6]. In avians, a HS imprint has been described in response to thermal conditioning where an initial HS exposure resulted in epigenetic modifications and an altered phenotypic response to a subsequent HS exposure [7], [8]. Notably, inheritance of embryonic HS-induced epigenetic alterations has been demonstrated in *Drosophila* [9]. However, the effect of *in utero* HS on mammalian epigenetic regulation later in life is largely unknown.

Direct HS impacts several biological parameters in domestic animals, and this response varies based on the duration and degree of the thermal challenge. Pigs are particularly sensitive to HS due in part to inadequate sweating capability leading to a limited capacity for evaporative cooling. Further, genetic selection for increased growth and lean body composition has resulted in an increased susceptibility to thermal stress [10]. A conserved mammalian response to both acute and chronic HS is reduced nutrient intake, presumably an attempt to reduce metabolic heat production. Interestingly, pigs reared in HS conditions typically have reduced skeletal muscle and increased adipose tissue mass [11]–[14]. Although the mechanisms responsible for HS induced alterations in body composition are not completely understood, it may be partially explained by our recent finding that basal insulin increased in a variety of HS models [15] including pigs [16]. Additionally, it was recently reported in a rodent model that HS stimulates insulin signaling in skeletal muscle [17]. The increase in insulin (a potent anabolic signal) occurs despite the marked reduction in feed intake and hyper-catabolic hormonal milieu that dominates during HS [1], [15].

The impact of HS during gestation on developing fetuses is mediated in part by alterations in metabolism and uterine blood flow, and both of these are suggested to cause postnatal phenotypic changes [18], [19]. Stressful intrauterine environments cause permanent deleterious effects on pig offspring growth and development [20]. In intrauterine growth retardation models, a validated model of *in utero* stress, piglets have altered skeletal muscle phenotypes, impaired intestinal development and reduced lifetime growth performance [20]–[22]. Moreover, it has been demonstrated that elevated maternal insulin levels [23], [24] and dietary restriction [25] alter offspring metabolic and hormonal profiles. Likewise, phenotypic modifications such as compromised development and altered physiological responses to HS later in life have been reported in fetal HS models for sheep [26], [27], rodents [2], [28] and poultry [29]. However, the effects of prenatal HS on offspring's future growth and metabolism have not been established in pigs. Therefore, the objectives of this experiment were to test our hypothesis that HS during gestation impacts postnatal body composition and thermoregulatory response to acute and chronic HS in pigs later in life.

## Materials and Methods

### Animals

All experiments involving the use of animals were approved by the Institutional Animal Care and Use Committees at Iowa State University and the University of Missouri.

### Experimental Design

A split plot study design was used to test the effect of gestational HS on the response to postnatal acute and chronic HS exposure. To accomplish this, pregnant gilts were exposed to four different environmental conditions during gestation, and resulting offspring were then exposed to thermal neutral [30] or HS environments during postnatal growth and development for either 24 h or 5 weeks.

### Gestational experimental treatments

Fourteen pregnant primiparous crossbred gilts (Large White x Landrace) were housed in one of four thermal environments throughout gestation at the University of Missouri Brody Environmental Chambers. Ambient diurnal temperature cycles were defined as TN (TN; 18 to 22°C) and HS (HS; 28 to 34°C) [31]. Gilts in TNTN (n = 4) and HSHS (n = 4) treatment groups were exposed to TN or HS conditions, respectively, for the duration of gestation. The remaining thermal treatment groups represent HS conditions only during the first half (HSTN, n = 3) or second half (TNHS, n = 3) of gestation. Thermal treatments began on day six of pregnancy and the mid-pregnancy switch for TNHS and HSTN groups occurred on day 55 of pregnancy. All dams were placed in TN conditions 10 days prior to estimated time of parturition. To avoid excessive maternal weight gain during gestation, all pregnant gilts were limit-fed 2.2 kg of a corn and soy based diet/day that met or exceeded maintenance and gestation requirements as determined by the National Research Council (NRC; 2012). Consequently, nutrient intake during gestation did not differ between gestation treatments. The dam's thermal response measurements were monitored as previously described [31].

Piglets were born naturally under TN conditions and remained with their original dams throughout the TN lactation period. Gestational thermal treatment did not affect litter size, piglet birth weight, piglet weaning weight, or male: female litter ratio (*P*>0.1, data not shown).

### Nursery period

Offspring were weaned (23±3 days of age) and transported to Iowa State University for postnatal analysis. Pigs were randomly assigned to group pens and provided ad libitum access to standard nursery phase diets and water for five weeks (until approximately 8 weeks of age). At the completion of the nursery phase, no gestational or gestational by time interactions were observed for piglet body weight (*P*>0.1, data not shown) or weight gain (*P*>0.1, data not shown). Thereafter, pigs assigned to the acute and chronic postnatal experiments were maintained in standard conditions for four and five additional weeks until initiating the acute and chronic postnatal HS experiments, respectively.

### Acute and Chronic Postnatal Experimental Treatments

To adequately understand the effect of HS experienced *in utero* on the postnatal response to HS, we tested the effect of gestational HS during both acute (24 h) and chronic (5 weeks) HS exposure. This approach allowed us to evaluate the initial and temporal HS response; when differences in acclimation and body composition parameters are more likely to be expressed. To this end, 96 offspring were selected from 14 litters for the acute and chronic postnatal experiments on the basis of weight gain and body weight. Offspring representing median weight gain and body weight from each litter were chosen to represent each dam with an equal number of males and females selected from each gestational treatment. Offspring from each litter were then randomly assigned to one of two constant environmental treatments: thermal neutral (TN, 21°C; 35–50% humidity) or HS (35°C; 24–43% humidity). Each room's temperature and humidity were continuously monitored by a data recorder (Lascar model EL-USB-2-LCD, Erie, PA), which continuously recorded environmental data in 30 min intervals. Each room's ambient temperature was controlled, but humidity was not governed.

For the acute postnatal HS experiment, 48 pigs (six males and six females per gestational treatment) were moved to individual housing at eight weeks of age. Pigs were acclimated under TN conditions during which time they were allowed ad libitum access to feed and water. Feed was formulated to meet or exceed the NRC (2012) recommended nutrient requirements for pigs at this age and weight. Prior to HS exposure, subcutaneous fat thickness (SFT) and longissimus dorsi cross-sectional area (LDA) at the 10^th^ rib were determined by ultrasound. At 12 weeks of age and during the lean tissue accretion phase, pigs were exposed to constant TN or HS conditions for 24 h. Pigs were moved into the TN or HS rooms in six blocks beginning at 0800 or 1100 h over three days. Each block consisted of eight pigs: one pig from each of four gestational treatments (TNTN, TNHS, HSTN, and HSHS) and two postnatal thermal treatments (TN and HS). For each block, respiration rate, rectal temperature, shoulder skin temperature, and feed intake were measured every 4 h during each 24 h period. Rectal temperature was measured with a ReliOn digital thermometer (Waukegan, IL). Skin temperature was obtained at the shoulder with a laser infrared thermometer (Extech Instruments Corporation, Waltham, MA). Respiration rate (breaths per minute; BPM) was determined by visual observation and calculated using a stopwatch. Body weight was obtained at the beginning and end of the 24 h period.

The remaining 48 selected offspring (six males and six females per gestational treatment) were utilized in the chronic HS experiment. Pigs were moved to individual housing and allowed to acclimate for two weeks in TN conditions during which time body weight and feed intake were determined weekly. At 14 weeks of age, half of the animals (six pigs per gestational treatment) were subjected to HS conditions while the remaining half remained in TN conditions. Throughout the five-week experiment, body weight and feed intake were measured weekly. Rectal temperature, respiration rate, shoulder skin temperature, and tail skin temperature were measured between 1400 and 1700 h twice weekly on all pigs. Additionally, ultrasound measurements of SFT and LDA were obtained on each pig at the beginning of the chronic experiment and after three and five weeks of TN or HS environment exposure. At the end of the acute and chronic treatment periods, pigs were sacrificed using a captive bolt followed by exsanguination.

### Metabolite and blood gas variable measurement

Blood was collected into lithium heparin and serum tubes upon exsanguination. Heparinized blood was assayed immediately using an iStat Portable Clinical Analyzer (Abbott Laboratories, San Diego CA). The iStat cartridge (CG8+) measured blood pH, carbon dioxide pressure, and concentrations of sodium, potassium, ionized calcium, glucose, hematocrit, hemoglobin, and carbon dioxide. Serum insulin and non-esterified fatty acid (NEFA) concentrations were measured at the end of the chronic postnatal treatment period by ELISA (Mercodia, Winston Salem, NC) and enzymatic colorimetric assay (Wako Chemicals, Richmond, VA), respectively (29).

### Ultrasound determination of subcutaneous fat thickness and longissimus dorsi area

To assess body composition, 10^th^-rib SFT and LDA were measured by ultrasound. Two 10^th^ rib images were collected by a certified technician using an Aloka 500V SSD ultrasound machine fitted with a 3.5 MHz, 12.5 cm, linear-array transducer (Corometrics Medical Systems Inc., Wallingford, CT). These measurements (SFT and LDA at the 10^th^ rib) are established indices and routinely utilized as a proxy for overall body composition in pigs [32]. Body weights were obtained at each ultrasound measurement for use as a covariate in the statistical analysis.

### Immunohistochemistry

Frozen longissimus dorsi muscle was cut into 10 µm sections. The muscle sections were then washed with phosphate buffered saline (PBS) for 10–15 min with agitation at room temperature. Tissue slides were then blocked with 5% bovine serum albumin (BSA) (solubilized in PBS) for 15 min at room temperature. The tissue sections were incubated with primary antibodies (laminin by Neomarkers REF RB-082-A; myosin heavy chain AF.951-a) diluted 1∶100 in 5% BSA overnight at 4°C. Tissue slides were washed with PBS 3×10 min with agitation at room temperature. Slides were incubated with secondary antibodies (Goat anti-rabbit rhodamine conjugated IgG, Millipore, Temacula CA; Goat anti-mouse fluorescein conjugated IgG, Millipore, Temacula, CA) diluted 1∶100 in 5% BSA in the dark. Slides were washed with PBS 3×10 min in the dark. Slides were blotted dry around the tissue sections and mounted with SlowFade Gold Antifade reagent with DAPI (Invitrogen, Carlsbad, CA) in the dark. Muscle sections devoid of primary and/or secondary antibody were used as negative controls.

### Microscopy

Microscopy was carried out using a Leica Microscope with the Q Capture Pro software (Surrey, BC, Canada) for fluorescence imaging. Raw images were converted to solid contrasting colors using Open Lab software (Perkin Elmer, Waltham, MA) and feret diameter was calculated using Image Pro Plus software (MediaCybernetics, Rockville, MD). Fiber typing was carried out using the Image J software by counting the number of fibers that exhibited type I MHC (presence of fluorescein).

### Statistical Analysis

All data were analyzed using the PROC MIXED procedure in SAS (SAS Institute, Cary, NC). Each model included day of sacrifice or block (if significant), gender, gestational treatment, postnatal thermal treatment, and the interaction between gestational and postnatal treatments as fixed effects. Dam nested in gestational treatment was used as a random effect. For temperature indices, feed intake, and body weight parameters acquired following HS, pre-HS measures were used as covariates. Body weight collected at the time of each ultrasound was used as a covariate in SFT and LDA analysis. For blood and growth parameters, the contrast between treatment groups exposed to TN compared to HS conditions for the first half of gestation was also measured and reported if significant. Statistical significance was defined as *P*≤0.05 and tendency as P≤0.1. All data is presented as LS-means ± SEM.

For repeated measures analyses of cumulative feed intake (during acute postnatal HS experiment) or weekly feed intake (during chronic postnatal HS experiment), rectal temperature, skin temperature, and respiration rate, two statistical models were utilized. For repeated measures analyses, the initial measurements obtained at the beginning of each experiment were used as covariates. For this reason, initial data points measurements were analyzed as described above using PROC MIXED. Also, for repeated analyses, each animal's respective parameter was analyzed using repeated measures with an auto-regressive covariance structure and time as the repeated effect. The model included gestational treatment, postnatal treatment, time, gender, and the interaction between gestational treatment, postnatal treatment, and time.

## Results

### Effect of Acute HS on Postnatal Temperature Indices and Performance

**Figure 1:**
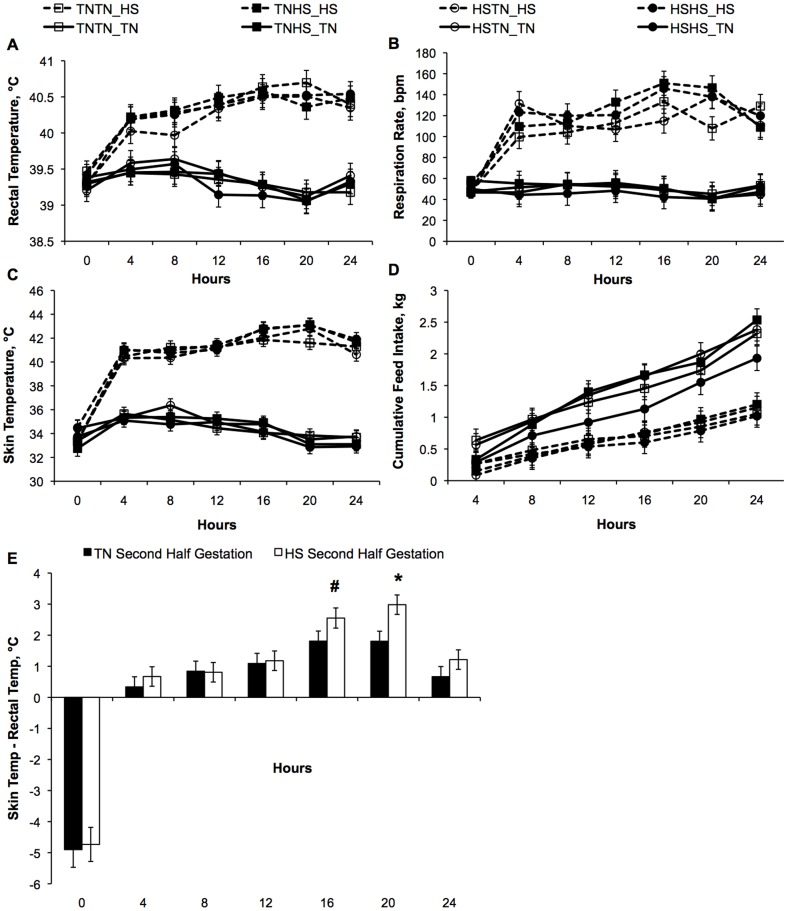
Acute postnatal heat stress (HS) alters temperature indices and feed intake over the 24 h treatment period. A) Rectal temperature increased during postnatal HS (*P<*0.001) and a postnatal treatment by time interaction was also detected (*P<*0.001). B) Respiration rate presented as breaths per minute (bpm) was elevated as a result of postnatal HS (*P<*0.001) in addition to the detection of a postnatal treatment by time interaction (*P<*0.01). C) Skin temperature was increased in pigs subject to postnatal HS compared to thermal neutral [30] counterparts (*P<*0.001) in addition to detection of a postnatal treatment by time interaction (*P<*0.001). A gestational by postnatal treatment interaction was observed in skin temperature (*P* = 0.05) whereby gestational treatments exposed to HS during the second half of gestation had higher skin temperature (0.5°C) under HS conditions but lower skin temperature under TN conditions (0.3°C) compared to HSTN and TNTN gestational treatments. D) Cumulative feed intake was reduced (*P*<0.01) in HS pigs compared to their TN counterparts. TN pigs gained weight while HS pigs lost weight over the acute HS period. No gestational effect was observed for feed intake or weight gain parameters (*P>*0.1). E) The difference between rectal and skin temperatures is elevated in pigs from TNHS and HSHS vs. TNTN and HSTN treatments at 16 h (*P = *0.1) and 20 h (*P = *0.01) of HS. ^#^ *P*<0.1; *P<0.05. Data shown represent the LS-mean ± SEM of n = 6 pigs per postnatal treatment per gestational treatment.

Regardless of gestational environment, all pigs had an immediate response to the acute postnatal HS treatment as rectal temperature (39.3 vs. 40.4±0.1°C), skin temperature (34.5 vs. 41.5±0.1°C), and respiration rate (49 vs. 122±4 bpm) were increased (*P*<0.001, Fig. 1A-C). Maximum temperature indices were achieved at 16–20 h post-initiation of HS, but the magnitude and temporal pattern of these temperature indices was not influenced by gestational treatments (Fig.1A-C). A gestational by postnatal treatment interaction was detected in skin temperature (*P* = 0.05, data not shown) whereby gestational treatments exposed to HS during the second half of gestation (TNHS and HSHS) had higher skin temperature (0.5°C) under HS conditions but lower skin temperature under TN conditions (0.3°C) compared to HSTN and TNTN gestational treatments. Cumulative feed intake was reduced 49% in HS pigs (*P*<0.01, Fig. 1D). Further, TN pigs gained weight while HS pigs lost weight (0.54 vs. −1.95±0.21 kg, *P*<0.01) over the acute HS period. No gestational effect was observed for feed intake or weight gain parameters. Further, the difference between skin and rectal temperature was larger at 20 h (3.0 vs. 1.5±0.5°C, *P* = 0.01) with a tendency in this direction at16 h (2.6 vs. 1.8±0.3°C, *P* = 0.1) in pigs from TNHS and HSHS than pigs from TNTN and TNHS (Fig. 1E).

### Baseline Postnatal Body Composition Parameters

**Figure 2:**
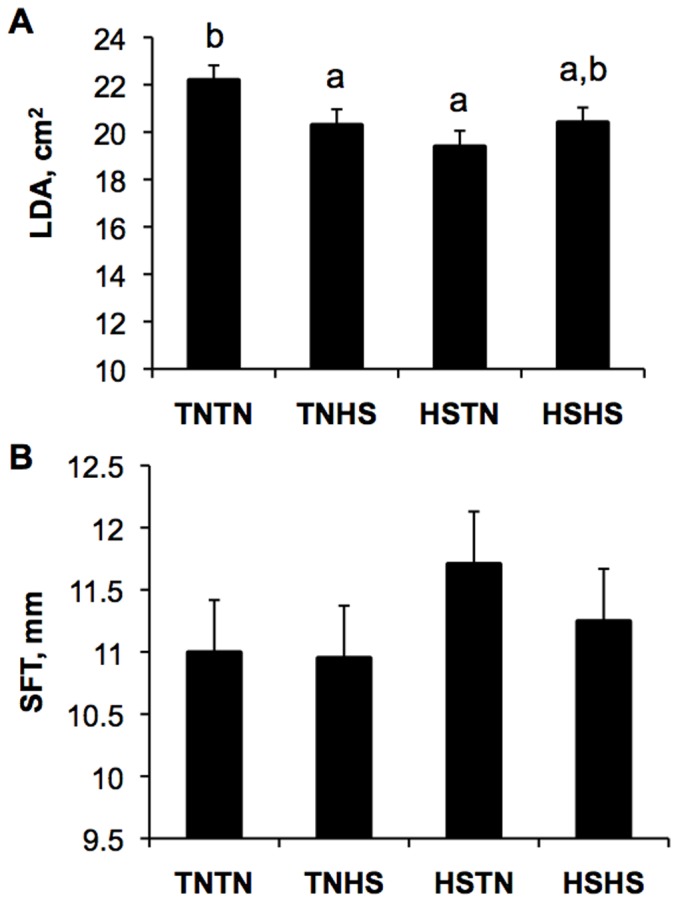
Effects of gestational thermal treatment on postnatal body composition indices at 12 weeks of age and prior to acute postnatal heat stress (HS). A) Longissimus dorsi cross-sectional area (LDA) is greater in pigs from TNTN dams compared to those from TNHS and HSTN dams, but not different than those from HSHS treated dams (*P = *0.03). B) No gestational effect is observed in subcutaneous fat thickness (SFT) at 12 weeks of age (*P = *0.58). Different letters indicate *P<*0.05.

At week 12 prior to the acute postnatal HS, LDA was larger in the TNTN group than TNHS and HSTN groups (9.3 and 14.5%, respectively, *P* = 0.03) and not different from the HSHS group (Fig. 2A). No gestational treatment effects were detected on SFT prior to postnatal HS (Fig. 2B).

### Effect of Acute HS on Postnatal Metabolic Profile

Regardless of prenatal treatment, acute HS altered several blood gas variables and metabolites (Table 1). Blood glucose concentrations (113.2 vs. 122.8±4.0 mg/dL) were elevated during acute HS whereas ionized calcium (1.38 vs. 1.33±0.02 mM), total CO_2_ (31.3 vs. 28.6±0.8 mM), and bicarbonate (29.8 vs. 27.2±0.8 mM) concentrations were decreased during HS conditions (TN vs. HS, *P*≤0.03). Concentrations of sodium and potassium, hematocrit, hemoglobin, and pH were unaffected by postnatal HS. Only blood potassium concentrations were influenced by gestational treatment such that any HS experienced during any stage of gestation decreased K concentration following acute HS (6.3, 5.5, 5.5 and 5.3±0.2 mM, *P = *0.04 for TNTN, TNHS, HSTN and HSHS, respectfully).

**Table 1:** Effect of gestational and acute postnatal HS on blood gas variables and metabolites.

	TNTN		TNHS		HSTN		HSHS			P		
Parameter	TN	HS	TN	HS	TN	HS	TN	HS	SEM	GestTrt^3^	PostTrt^4^	G x A^5^
Na, mM	141	140	142	141	141	141	141	142	1	0.36	0.89	0.58
K, mM	6.2^b^	6.5 ^b^	5.1^a^	5.8 ^a^	5.6 ^a^	5.4 ^a^	5.2 ^a^	5.5 ^a^	0.3	0.04	0.26	0.56
iCa, mM^1^	1.35	1.27#	1.39	1.37#	1.40	1.36#	1.38	1.33#	0.03	0.09	0.02	0.85
Glucose, mg/dL	111	133#	110	125#	115	111#	117	122#	8	0.78	0.03	0.18
Hematocrit, percent PCV^2^	36	36	36	36	37	37	36	38	1	0.57	0.73	0.90
Hemoglobin, g/dL	12.3	12.2	12.1	12.2	12.6	12.5	12.3	12.8	0.4	0.59	0.70	0.90
pH	7.345	7.392	7.361	7.355	7.337	7.409	7.396	7.403	0.036	0.69	0.25	0.68
CO_2_ Pressure, mmHg	51.3	49.6	54.1	47.5	59.6	45.9	47.2	44.2	4.9	0.55	0.08	0.62
Total CO_2_, mM	30	28#	32	28#	34	30#	30	28#	12	0.38	0.01	0.58
HCO_3_, mM	28.2	26.8#	30.7	26.3#	32.0	28.8#	28.1	27.0#	1.6	0.37	0.01	0.57

1 iCa, ionized calcium.

2 PCV, packed cell volume.

3 GestTrt, gestational treatment.

4 PostTrt, postnatal treatment.

5 G x A, interaction between gestational and postnatal treatment groups.

Different letters indicate GestTrt comparisons P<0.05.

# indicates PostTrt comparisons P<0.05.

### Effect of Chronic HS on Postnatal Temperature Indices and Growth

**Figure 3:**
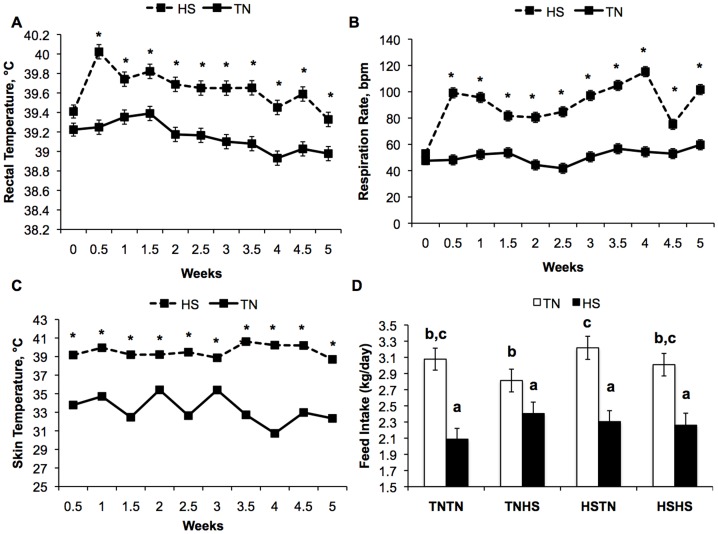
Chronic postnatal heat stress (HS) alters temperature indices and feed intake over the five-week treatment period. A) Rectal temperature is elevated by postnatal chronic HS (*P<*0.001) and postnatal treatment interacts with time (*P<*0.05). B) Respiration rate, breaths per minute (bpm), is elevated in HS compared to thermal neutral [30] postnatal treatment (*P<*0.001), and a postnatal treatment by time interaction was also observed (*P<*0.001). C) Skin temperature was elevated as a result of postnatal HS (*P<*0.001) in addition to a postnatal treatment by time interaction (*P<*0.001). D) Feed intake is reduced in HS compared to TN pigs (*P<*0.001), and a gestational by postnatal interaction was observed where pigs from all gestation treatments had similar feed intake during HS conditions, but under TN conditions, pigs from HSTN treated dams had greater feed intake than those from TNHS dams, but not different than those from gilts subjected to HSHS or TNTN gestational treatments (*P = *0.04). Data show the mean ± SEM of n = 6 pigs per postnatal treatment per gestational treatment. * indicates *P<*0.05 for postnatal treatment by time comparisons. Different letters indicate *P<*0.05 for gestational by postnatal treatment comparisons.

Over the five-week postnatal treatment period, rectal temperature (39.1 vs. 39.7±0.04°C), skin temperature (33.3 vs. 39.6±0.1°C), and respiration rate (51 vs. 94±2 bpm) were increased (*P*<0.01) in HS animals (Fig. 3A-C), but these body temperature indices were not influenced by HS during gestation. During chronic postnatal HS, a temporal effect of postnatal treatment (*P*≤0.05) but not gestational treatment (*P>*0.05) was observed in temperature indices over the five-week period (Fig. 3A-C).

Pigs in chronic HS conditions had reduced body weight gain during the five-week period compared to TN pigs (35.4 vs. 25.9±0.8 kg, *P<*0.01). A postnatal environment by gestation treatment interaction was observed in feed intake (*P*<0.04) in that pigs from all gestation treatments had similar feed intake during HS conditions, however under TN conditions, pigs from HSTN dams had greater feed intake than those from TNHS dams, but feed intake was not different than those from gilts subjected to HSHS or TNTN gestational treatments (Fig. 3D). Feed intake was positively correlated with SFT (R = 0.87, *P<*0.001), LDA (R = 0.41, *P<*0.01), and final body weight (R = 0.82, *P<*0.001). Feed efficiency (kg feed/kg body weight gain) was not affected by gestational or postnatal treatments (data not shown).

### Effect of Chronic HS on Postnatal Body Composition Parameters

**Figure 4:**
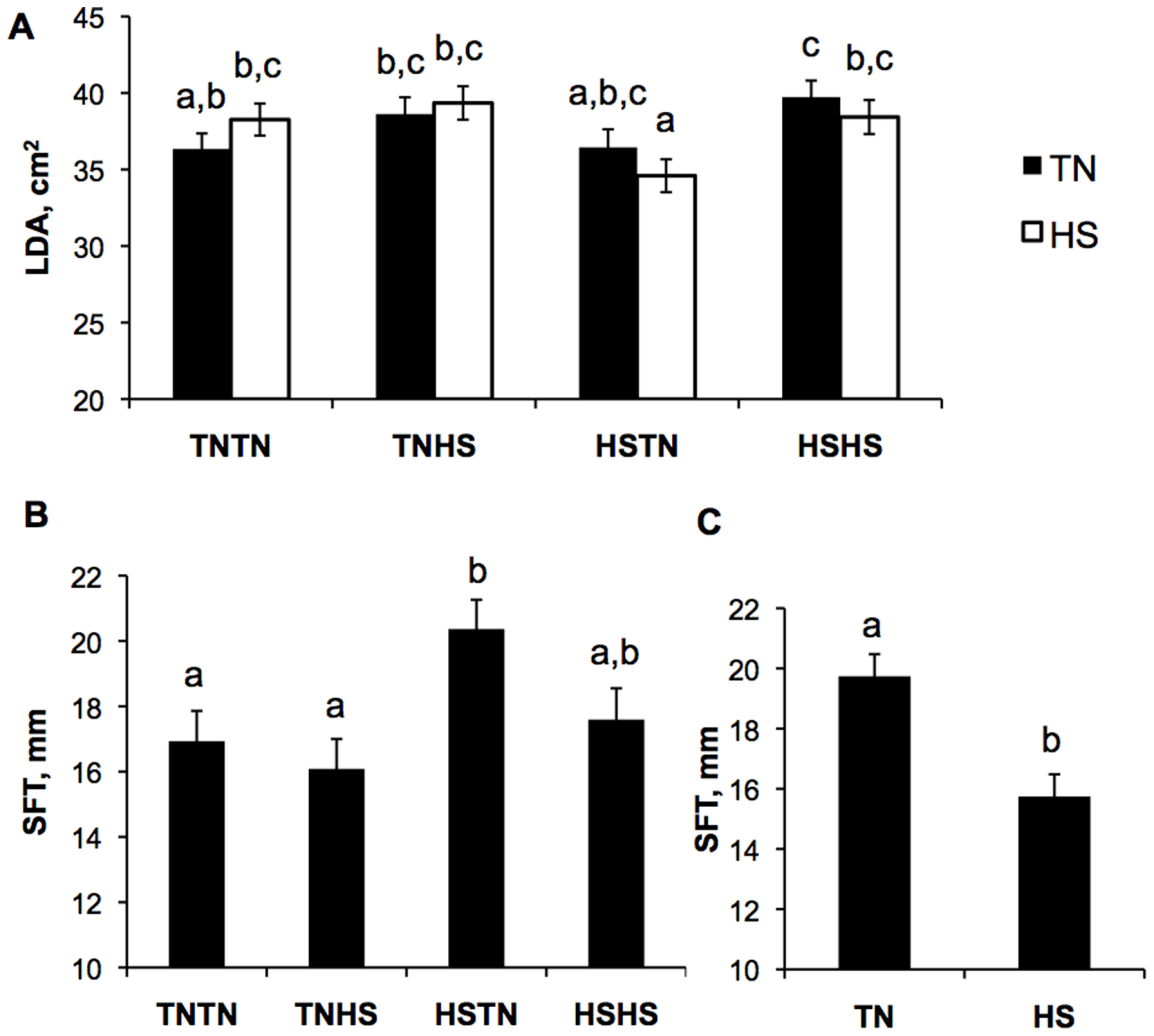
Effects of gestational and chronic postnatal heat stress (HS) on body composition indices following chronic (5 weeks) HS exposure (19 weeks of age). A) An interaction was observed between gestational and postnatal treatments on longissimus dorsi cross-sectional area (LDA) (*P = *0.045), and a tendency was observed for gestational treatment to have an effect on LDA (*P = *0.07). B) HSTN pigs have greater subcutaneous fat thickness (SFT) than TNTN and TNHS pigs while HSHS pigs are not different from other gestational treatments (*P = *0.04). C) Chronic postnatal HS resulted in a reduction in SFT, regardless of gestational treatment (*P = *0.002). Data show the mean ± SEM of n = 6 pigs per postnatal treatment per gestational treatment. Different letters indicate *P<*0.05.

An interaction was observed between gestational and postnatal treatments in LDA at the end of the chronic treatment period (*P* = 0.05, Fig. 4A). The interaction demonstrated five weeks of HS during weeks 14–19 of age resulted in altered LDA compared to TN counterparts differentially in pigs from dams subjected to HS during the first half or second half of gestation. Interestingly, an overall gestational effect was also observed for SFT (*P* = 0.037, Fig. 4B) in which HSTN pigs had greater SFT than TNTN and TNHS pigs while SFT of pigs from HSHS dams was not statistically different from other groups. Further, irrespective of postnatal HS, pigs from dams that experienced HS during the first half of gestation (HSHS and HSTN) had increased (13.9%) SFT compared to pigs from dams exposed to TN conditions during the first half of gestation (18.9 vs. 16.6±0.7 mm, *P* = 0.05; Fig. 4B). Unlike LDA, chronic postnatal HS decreased SFT by approximately 20% (*P*<0.01), regardless of gestation treatment (Fig. 4C).

### Longissimus Dorsi Fiber Type Analysis

IHC was performed on the LDA samples collected from pigs reared in the TN conditions postnatally at 19 weeks of age using antibodies specific for laminin and myosin heavy chain. A prominent intensity of fluorescein signal within the fiber indicated the presence of type I myosin heavy chain. In each treatment, the percentage of type II fibers significantly outnumbered type I fibers (TNTN, 88.7±2.4% type II and 11.3±2.4% type I; HSTN, 92.9±2.7% and 7.1±2.7%; TNHS, 91.8±2.7% and 8.2±2.7%; HSHS, 88.9±2.7% and 11.1±2.7%). Collectively, gestational treatment did not affect (*P* = 0.7) fiber type distribution in longissimus dorsi samples.

Feret diameter was used to determine the size of all fibers within the LDA samples. The average size of muscle fibers was similar (*P* = 0.39) across treatments (48.2±4.1 µm for TNTN; 41.5±4.6 µm for HSTN; 42.3±4.4 µm for TNHS; 47.9±4.5 µm for HSHS) indicating development of a consistent muscle fiber size in all treatments.

### Effect of Chronic HS on Postnatal Metabolic Profile

**Figure 5:**
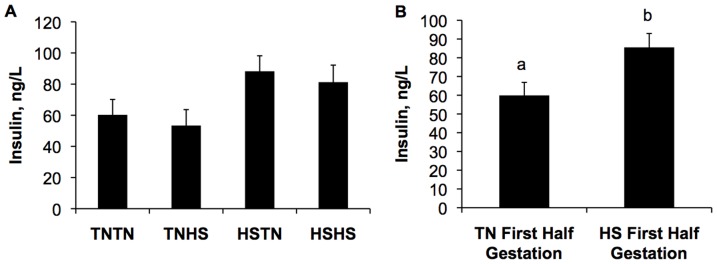
Effect of gestational treatment on circulating insulin concentrations at the end of the chronic treatment period, regardless of postnatal thermal treatment. A) Gestational treatment had a tendency to affect circulating insulin concentrations (*P = *0.0.09). B) Circulating insulin concentrations were elevated in pigs from dams exposed to heat stress (HS) conditions during the first half of gestation compared to those from dams gestated in thermal neutral [30] conditions during the first half of gestation (*P = *0.01). Different letters indicate *P<*0.05.

Compared to TN counterparts, postnatal HS increased (*P*<0.05, Table 2) blood pH (7.330 vs. 7.390±0.011) and reduced (*P*<0.05) circulating glucose (99.3 vs. 93.9±2.0 mg/dL), hematocrit (41.1 vs. 38.2±0.5% PCV), hemoglobin (14.0 vs. 13.0±0.2 g/dL), CO_2_ pressure (60.0 vs. 51.0±1.2 mmHg), total CO_2_ (33.5 vs. 32.0±0.4 mM), and bicarbonate (31.7 vs. 30.6±0.4 mM; Table 2). Irrespective of postnatal treatment, there was a tendency for a gestational effect on blood insulin concentrations at the end of the chronic HS period (*P = *0.09, Fig. 5A). Further, pigs from gilts heat-stressed during the first half of gestation had increased (33%; *P* = 0.01, Fig. 5B) blood insulin levels when compared to pigs from gilts exposed to TN conditions during the first half of gestation. There was no postnatal treatment effect or gestational by postnatal treatment interaction detected on circulating insulin concentrations. Additionally, no gestational or postnatal treatment effects were observed for NEFA concentrations (Table 2).

**Table 2:** Effect of gestational and chronic postnatal HS on blood gas variables and metabolites.

	TNTN		TNHS		HSTN		HSHS			P		
Parameter	TN	HS	TN	HS	TN	HS	TN	HS	SEM	GestTrt^4^	PostTrt^5^	G x P^6^
Na, mM	140	139	141	139	141	140	141	141	1	0.40	0.06	0.65
K, mM	6.3	6.4	6.1	6.2	6.8	6.8	5.9	6.6	0.3	0.25	0.29	0.65
iCa, mM^1^	1.36	1.35	1.36	1.35	1.35	1.39	1.37	1.36	0.18	0.81	0.89	0.47
Glucose, mg/dL	105	95#	96	95#	96	93#	101	92#	4	0.66	0.03	0.50
Hematocrit, percent PCV^2^	42	38#	40	38#	42	40#	40	36#	1	0.12	<0.01	0.74
Hemoglobin, g/dL	14.2	13.0#	13.8	13.1#	14.3	13.5#	13.7	12.4#	0.3	0.13	<0.01	0.76
pH	7.316	7.417#	7.335	7.395#	7.320	7.361#	7.348	7.387#	0.021	0.63	<0.01	0.33
CO_2_ Pressure, mmHg	63.6	48.8#	58.4	48.8#	60.6	55.5#	57.4	50.9#	2.2	0.30	<0.01	0.24
Total CO_2_, mM	34	33#	33	31#	33	33#	33	32#	1	0.24	<0.01	0.65
HCO_3_, mM	32.5	31.3#	31.0	29.7#	31.4	31.0#	31.6	30.4#	0.7	0.91	0.04	0.91
NEFA, µeq/L^3^	96.7	70.7	73.7	81.2	68.6	78.8	92.2	78.0	16.3	0.87	0.62	0.62

1 iCa, ionized calcium.

2 PCV, packed cell volume.

3 NEFA, Non-essential fatty acids.

4 GestTrt, gestational treatment.

5 PostTrt, postnatal treatment.

6 G x A, interaction between gestational and postnatal treatment groups.

# indicates PostTrt comparisons P<0.05.

## Discussion

We and others have previously demonstrated altered body composition and metabolic profile in response to HS in a variety of animal models [15], including pigs [16]. Heat stress experienced during fetal development can result in physiological anomalies that extend into post-gestational life [1]–[3]. Models involving alterations in maternal metabolic environment and nutritional status have also been demonstrated to negatively impact lifetime growth and development of offspring in numerous species including humans [23], [33]–[36]. However, the impact of *in utero* HS on offspring with respect to metabolic programming and response to HS later in life has not been previously reported in pigs. The objective of this experiment was to test our hypothesis that HS during gestation alters the growth variables and the metabolic profile of offspring during acute and chronic HS challenges.

Our hypothesis is supported in that these data demonstrated prenatal HS altered offspring postnatal body composition. Alterations in LDA (used to predict whole body composition) due to gestational treatment were first observed at 12 weeks of age and were maintained through 19 weeks of age when the study ended (Fig. 2A and Fig. 4A). Essentially, piglets produced from HSTN treated gilts had the smallest LDA whereas pigs produced from TNTN treated gilts had the largest LDA. Additionally, the effect of chronic HS exposure during postnatal growth on LDA was influenced by the gestational stage HS was experienced by their dams during prenatal development. Pigs produced from gilts heat-stressed for the first half of gestation (HSTN and HSHS) had reduced LDA following postnatal HS whereas TNHS and TNTN gestational treatment counterparts had increased LDA following chronic postnatal HS.

Prenatal stress has previously been shown to impact muscle differentiation and development in intrauterine growth restriction (IUGR) models where muscle primary and secondary fiber number, weight, and cross-sectional area are reduced as a result of IUGR [20], [26], [37]–[40]. Importantly, IUGR was observed to impact expression of genes related to myogenesis as early as day 30 of gestation [41] supporting our observations that *in utero* HS during the first half of gestation resulted in phenotypic differences in LDA of offspring. Similar to the IUGR model, maternal nutrient restriction during early embryonic development alters muscle fiber number in offspring across multiple species [34], [42], [43]. Maternal nutrient restriction during early but not mid and late gestation resulted in alterations in muscle fiber development [35]. Conversely, maternal nutrient restriction applied during late gestation resulted in decreased muscle weight shortly after birth. Importantly, in our experiment, maternal nutrient intake during gestation did not differ between treatments so the results described herein are directly related to the consequence of environmental-induced hyperthermia.

These differential effects of stress occurring in early vs. late gestation on muscle development could be explained by the temporal pattern of muscle fiber formation throughout gestation. Hyperplasia of primary and secondary fibers ceases at an average of 35 and 90 days of gestation in pigs, respectively [44]. Secondary fiber formation is most susceptible to gestational stress, and fiber number limits muscle mass capacity later in life [44]. Taken together, the literature coupled with our data suggests gestational HS during the first half of gestation (i.e. prior to day 55) results in impaired primary muscle fiber development causing permanent and negative effects on muscle growth. Although gestational HS did not affect fiber type distribution nor fiber size within the LDA, a phenotypic difference as a result of gestational HS may have been more likely observed in the total number of cells within the whole LDA.

Although the effects of maternal nutrient restriction and IUGR stress on offspring muscle development are well-defined, a strong understanding of the implications gestational HS has for progeny is significantly lacking. Maternal HS in sheep has been shown to reduce uterine blood flow resulting in nutrient restriction and decreased placental size where HS applied late in gestation was accompanied by a reduction in uterine blood flow and resulted in reduced fetus weight and muscle protein [27]. Interestingly, in chicks exposed to HS during early post-natal life, the percentage of muscle mass decreased immediately following HS, although by 42 days of age, was greater than control chicks [45]. Taken together with our data, it appears HS applied during early development may alter muscle fiber development with implications for postnatal muscle development. Moreover, the effect of maternal HS on offspring development likely shares common mechanisms with IUGR and maternal nutrient restriction models.

Not only did gestational HS appear to impact muscle mass, but implications for the adiposity of progeny also exist. The gestational treatment group with the smallest LDA (HSTN) also resulted in the production of offspring with the largest SFT (Fig. 4B, 3C). The HSTN group also had highest feed intake over the chronic part of the experiment, and feed intake was strongly correlated to adiposity, thereby providing a potential explanation for SFT differences. Further, pigs exposed to *in utero* HS during early gestation had greater SFT than those exposed to TN conditions during early gestation. There is accumulating evidence in the human literature to suggest a role for gestational environment on offspring body fat and obesity. In studies of the 1944–1945 Dutch famine, children from women exposed to nutrient restriction during early gestation, but not mid and late gestation, have increased prevalence of obesity [33]. Animal studies have helped to define a causal relationship between maternal stress and offspring adiposity [46], [47]. A critique to our observations of changes in body composition parameters is the limitation of SFT measurement to one anatomical location. Subcutaneous fat thickness corresponds to overall body composition in normal commercial pigs [32], however, it is unknown if maternal HS alters fat depot partitioning. Investigations to determine effects of HS on adipose tissue depot partitioning and whole body composition would provide a better understanding of the mechanism by which this observation occurs.

The shift toward increased adiposity and decreased lean body composition of progeny subjected to *in utero* HS during early gestation was accompanied by increased circulating insulin concentrations in offspring at the end of the chronic HS period (Fig. 5). We and others have reported increased circulating insulin concentrations [15], [16] and signaling [17] in response to acute postnatal HS in a variety of models. While the reason for enhanced circulating insulin during HS response remains unclear, it likely includes insulin's role in activating the cellular stress response [48]. In contrast to our previous experiments [15], [16] we did not observe an increase in circulating insulin concentrations in response to chronic postnatal HS. Reasons for this are not clear but may be partially explained by higher circulating insulin levels in progeny from dams subjected to HS during the first half of gestation (Fig. 5B). Additionally, our experimental design did not include a pair-fed thermal neutral group of pigs so comparing circulating insulin in animals on markedly different planes of nutrition is complicated by differences in nutrient intake. Regardless, what remains to be determined is if elevated insulin concentrations in pigs derived from HSTN and HSHS gestational treatments is causative for the increase in adiposity or is an independent observation. From an epigenetic programming perspective, maternal or paternal insulin dysregulation alone has been demonstrated to alter offspring response to insulin [23]. Likewise, in humans an *in utero* diabetic environment increases risk of diabetes in offspring [24]. Similar findings have been reported in response to IUGR and nutrient restriction during early gestation with alterations in insulin signaling in offspring [36], [49]. Collectively, these data suggest that HS may alter metabolic programming of piglets occurring during the first half of gestation subsequently impacting the physiological insulin response during postnatal HS.

We have previously demonstrated HS-induced alterations in insulin concentrations and body composition; however, the effect of HS experienced *in utero* on these characteristics has not been previously reported in pigs. In summary, the offspring from dams subjected to HS during the first half of gestation had smaller LDA following chronic postnatal HS compared to those offspring from dams gestated in TN conditions during the first half of gestation. Additionally, pigs from dams heat-stressed for the first half of gestation had increased SFT and circulating insulin concentrations compared to pigs produced from sows exposed to TN conditions for the first half of gestation. Moreover, postnatal feed intake was altered by gestational treatment and positively correlated to SFT. The aforementioned phenotypic changes are a direct result of environmental heat stress as maternal nutrient intake (during gestation) was similar between thermal environments. Our data suggest programming of piglets may occur *in utero* during the first half of gestation resulting in an altered metabolic hormone profile and body composition during subsequent growth and development. Additional investigations will help elucidate the mechanisms regulating the gestational HS-induced alterations in metabolic profile, muscle development, and lipid accretion.
